# What Is Mucus? What Is Pus?

**Published:** 1868-11

**Authors:** H. R. Noel

**Affiliations:** Professor of Physiology.


AMERICAN JOURNAL

OF

DENTAL SCIENCE.

Vol. II. THIRD SERIES—NOVEMBER, 1868. No. 7.

### ARTICLE I.

### *What is Mucus? What is Pus?*

Review Lecture, Delivered at the Baltimore College of Dental Surgery.
By H. R. Noel, M.D. Professor of Physiology.

We will this morning, give you a rapid *resumé* of the last three lectures, and endeavor to fix more firmly in your minds the recent views which obtain, as regards Mucus and Pus.

The old theories are being ignored, and Virchow, Paget, Beal, and others, have revolutionized physiology and are fast revolutionizing medicine, it is expedient therefore that we acquire an accurate knowledge of these new views, for they have an important bearing upon our present course.

You remember that we said the skin and mucus membranes were nearly identical, that anatomically and physiologically they were so very nearly the same, that we would for the present discard any difference. Analogous in formation they are analogous in function. As regards the anatomical elements we find the following—

1. Areolar Tissue.
2. Basement membranes.
3. Nuclei, nuclear matter, germs, &c.
4. Larger nuclei and cells forming and spherical masses.
5. Fully formed cells.
6. Epithelial cells, and Epidermic Scales.

Upon the skin we have *dried flakes*, they are dessicated cells, mere scales, lifeless. Upon the mucus membrane they retain their moisture and have a definite form, they are moist cells, we call them Epithelium.

Now beginning at the epithelial cells, or at the epidermic scales, and going downward we have the same anatomical elements, but in an inverted order: (1) Scales or Cells. (2) Cells and Spherical masses. (3) Spherical masses and nuclei. (4) Nuclear matter, nuclei, granules &c. (5) Basement membrane. (6) Areolar derm, or areolar tissue subjacent.

Beneath this basement membrane and in the areolar tissue, ramify blood vessels of extreme minuteness, supplying pabulnm for the surrounding structures, and furnishing by osmotic action through the basement membrane, abundant food for the broods of young cells constantly developing from the outer or upper surface of this membrane. Upon this membrane we have plasma and germs, cytoblasts or nuclei, nuclear matter; these latter forming, use up the plasma and draw fresh supplies from the vessels beneath.

These are primordial anatomical forms, and are common to all tissues everywhere; from just such elements all tissues are developed—i e from these germs or nuclei, with the plasma of the blood as a pabulum. Upon the mucous membranes, they form epithelium; upon the skin, they form and become dried, flattened, shapeless scales, and when adherent laterally they often exhibit a sort of membrani-form expansion, and we call this the epidermis or cuticle; you have often seen it raised in blistering.

The epithelium and the epidermis are so nearly the same that we are justified in calling them one, they are of a common parentage, common history, similar growth and development; one happens to be retained moist and keeps its shape, and the other by accident of position is dessicated, shrunken shapeless. They both moult constantly and are as quickly replaced by the young cells forming near the basement or limiting membrane. Their functions are more mechanical than otherwise, being protective of softer tissues beneath, and in this function the skin and mucous membrane also participate, though they have other and far higher functions, to be hereafter described.

Following Dr. L. S. Beale, the great microscopist of London, we will endeavor to enter that nebulous land "the first dawn of vitality" and trace the progressive developments. Dr. Beale is here most happy in his descriptions, plain, simple, clear and comprehensive, he leaves no doubt or obscurity as to his views.

In a perfectly transparent nutrient fluid, resting upon the basement membrane, the phenomena occur in the following sequence:— (1) Infinitesimally small granules appear. (2) These at points begin to coalesce and to form dots and granular collections, gradually enlarging. (3) Growing still larger, they become minute masses, and show a molecular motion among the granules. (4) Increasing still, they show not only the motion among the particles, but little currents in the nutrient fluid, and these faintly marked currents move perceptibly *to* and *from* the centres of these little masses, and we can now see that these masses are increasing in size, not by *accretion* merely, not by mere deposits upon the circumference, but in virtue of some power working at the centre.

This central power is the controling one, and the growth is regulated and the currents determined by it.

Dr. L. S. Beale thinks, and also Dr. Chambers, that this is "the first dawn of vitality," that up to this point it is impossible to tell whether the granules are moving in obedience to a physical or to a vital force, but that this point is settled, and conclusively so, when we perceive currents moving to and from the centres of the masses, and masses increasing not by peripheral accretion but by growth from the centre to its circumference. These clear, transparent, growing masses, are called masses of germinal matter, and they are the same as the *germs*, cytoblasts, nuclei, &c., of the older physiologists,having the jelly like consistency, and in fact resembling minute *specks* of jelly, or minute *balls* of jelly, granular and in constant motion.

(5) These masses of germinal matter increase in number and with great rapidity, and the increase is by two distinct methods (a) by the method of growth just described, (b) by a species of dividing, budding segementation &c. By this latter process, each parent mass may divide into two or even four smaller masses, these latter portions inheriting to a certain degree, the power or vital force of the parent mass. Now these secondary masses may also take an inclination towards propagation and under suitable conditions may run to the 20th, 30th, or even 100th generation possibly. Remember, however, that this always take place at the *expense of vital force,* and soon reaches a point where vitality ceases and reproduction of course stops.

Upon the basement membrane therefore, we have these masses of germinal matter and it is with them we will have to deal.

You perceive that so far, we have given germinal matter but two powers (1) power of growth, which involves the power of assimilation. (2) power of reproduction or propagation. Now there is a third power, it is the power of development, that in virtue of a high degree of vital force, germinal matter has the power of changing into tissue, into what Dr. Beale calls " formed material," and this change into formed material or true tissue, is the highest expression of vital force that germinal matter can exhibit. The third power, or power of development, is the highest of the three.

Once having attained the high degree of " formed material," it never again returns to the state of germinal matter, or exhibits any of the vital phenomena, which belong to and characterize this state. Let us be very explicit, and we therefore repeat; that when this third and highest power of germinal matter is once exhibited and formed material results, that this formed material never again assumes any of the powers or functions of germinal matter, but as regards these it is *quiescent;* it may have other and higher functions as we shall see in muscular and nerve tissue, but it never again comes within the domain of that class of functions, which we designate as nutritive or vegetative, embracing the three powers assigned to germinal matter.

If therefore formed material have vitality at all, we do not see it in activity, but only in its passive resistance to chemical forces, which unbalanced would soon return it decomposed, to its primitive elements of C. H., N. O., &c.

Formed material may possess *latent vitality*, germinal matter is characterized by its activity and is never quiescent except under duress.

But this will be more fully discussed hereafter, when we investigate the physical and vital properties of tissues.

We will say that germinal matter corresponds to the *germs*, cytoblasts, nuclei, &c., of the old physiologists, in being the centres of vitality foci of nutritive changes, &c.

The vital force is exhibited under the three forms given. (1) Assimilative change. (2) Propagation. (3) Development. By the 1st power, we mean that germinal matter has the power of changing the elements of a nutrive fluid, so that they shall no longer be different from, but shall in every particular be the same as the germinal matter itself, and thus the germinal matter can *grow*, by incorporating this changed and transformed pabulum with the mass of growing matter. The 2nd. power, or power of propagation, is simply one of subdivision of the parent masses, and the transmitting to the offspring, or these secondary masses, of the 1st. and 2nd. powers, they therefore present the same phenomena. But in the extreme exhibition of the 2nd. power all individuality seems to be lost, and the masses acquire the same size, shape, outline and appearance, as if each were a mere repetition of the other.

In the exhibition of the first power, or rather in the first phenomena attending the initial stages, the masses are irregular in shape, size and appearance, and possess more or less individuality, which as above described, diminishes in exact ratio to the exhibition of the 2nd. power. The 3rd. power or the developmental, gives us tissues, gives us the formed material of Dr. Beale, and here the vital force ceases to be active.

We are struck by the fact, yet it is but a repetition of the same fact as regards the 2nd. power, only in the latter case the vital force is slowly exhausted by extreme subdivision, in the 3d. it ceases abruptly with the development.

Now to return to the " centres of vitality" i e these masses of germinal matter upon the membrane, with the blood vessels containing nutrient plasma just beneath; do you not see that we have all the conditions necessary for the exhibition of the three powers? The germinal matter increases in size, increases in amount, and undergoes development. The development, or exhibition of the 3d. and highest power, is seen in the production of the epidermic and epithelial cells, the nucleus of the cell is the germinal matter, the cell wall is formed material; often the contents of the cell are also formed material. These cells never beget other cells, these cells never assume any functions belonging to the germinal matter, they are formed material, and therefore inactive.

Mark well, that the highest exhibition of vital power, the typical health of a mucous membrane or of the skin, is found where these masses of germinal matter develope slowly and regularly into epidermic and epithelial cells for the normal protection of the parts subjacent.

*The normal functions being thus obtained by the normal development of these masses, we would suppose the structures to be in a state of typical health. Germinal matter upon the basement membrane, can give no higher exhibition of vital force, than to develope into normal epidermic or epithelial structures.*

*In doing this it fulfils accurately its functions, it carries out the design of the Supreme Architect. This then being the highest exhibition of vitality possible, any deviation from it must of necessity be in the direction of lowered vitality.*

Now we come to the question of Mucus and Pus, and you at once appreciate the scale in which these two substances must be placed.

Both mucus and pus are often—very often found upon mucous membranes, but it is not the function of the mem brane to secrete either mucus or pus. So far from this being the case, so far from the membrane, normally producing mucus it is absolutely a sign of lowered vitality to find it there; it is an evidence of impaired function. A membrane producing mucus is not in a state of typical health, and a membrane producing pus, is but one single degree more advanced in disease. Contrary therefore to all our past experiences contrary to the teaching of the schools in the past, and allowed now, only from overwhelming evidence, we are forced to acknowledge that mucous membranes never secrete mucus unless they are diseased, and pus is only one degree lower, and a product of the same process.

Germinal matter has three powers, the 3d. or highest is found in the normal development into tissue, now if we exclude that power we have only two left. What are the relations of mucus and pus to these two? There are agents which depress the vital power of the whole organism, and there are some which seem to exert their baneful influence more especially upon mucus membranes, cold is one belonging to this latter class. We are all familiar with the annoying flow as a result of cold acting upon the schneiderian membrane of the nose; we term it cold in the head.

Under the influence of cold, the 3d. or highest power of germinal matter seems to be neutralized or to become inoperative, if not entirely yet partially so, while the 1st. and 2nd. powers continue in full play; in fact when the 3d. ceases to be exhibited, the other two seem to be often most active. It seem as if any agent which will lower the vitality of germinal matter, is capable of producing a mucus flux, and that if the depression be carried to any considerable extent the next stage is a purulent flux.

An agent acting moderately, will give a mucus discharge i e a blennorrhœa ; one acting with more intensity will give a purulent discharge, i e a pyorrhœa.

Dr. Beal, Chambers, &c., have given most admirable description of the two processes, or rather the continuation of the one process through the two stages of mucus and pus.

If from a fresh mucuous surface, a small portion of recent mucus be taken and kept warm, while it is carefully examined under a microscope, the following phenomena will be observed.

(1) Granules and masses of germinal matter forming.

(2) Increasing in size and dividing, budding &c.

(3) A clear substance gradually appears between the masses of germinal matter, surrounding and separating them from each other.

(4) As the masses decrease in number this clear substance increases in quantity.

(5) The fresher the mucus, and the nearer it is obtained from the membrane, the more there is of this transparent substance and the smaller the number of masses of germinal matter.

(6) The older the mucus and the further it is from the membrane, the smaller the quantity of transparent substance and the larger the number of masses of germinal matter, until at some little distance from the membrane there is no longer found any transparent substance, but innumerable masses of germinal matter.

The highest form of mucus is that which possesses the largest amount of this clear transparent medium and the fewest number of these masses of germinal matter. The lowest form is that where little or no transparent substance is seen, but large numbers of masses of germinal matter. The transparent substance or medium lying around and connecting the masses of germinal matter, called *mucin*, is remarkable for its clearness, and transparent, adhesive, stringy character.

The history of its production seems to be as follows: the globules or masses of germinal matter, though unable to give full expression to the 3d power, make an abortive attempt to do so, and give mucin as formed material, this though somewhat of a failure, is yet an effort in the right direction and marks a fair degree of vitality in the germinal matter.

Dr. Beale has proven this mucin to be formed material and analagous to cell walls &c., for under "the carmine fluid" the masses of germinal matter are dyed but the mucin is scarcely if at all effected by it; the test of formed material being its indifference to the fluid above mentioned. Remember that germinal matter is *invariably*, and formed material *never* dyed by "the carmine fluid," so in treating any specimen of mucus we must estimate the vitality of the germinal matter, from which we get mucin, by the amount of mucin, or formed material which we find present. You can even form a pretty accurate estimate of the condition of the skin or of a mucous membrane, by a careful observation of the secretion found upon it; this of course refers to such instances as may occur where we have the opportunity of a critical microscopical examination. If merely moist or with a small amount of clear non-adhesive fluid the condition is favorable :—if a clear tenacious mucus be present the condition is still favorable :—if an opaque, cloudy, non-adhesive fluid with no mucin, but myriads of masses rapidly propogating and increasing in quantity, then indeed we prognosticate evil, for this is pus and the process is called suppuration.

Here the germinal matter has vital force sufficient, only for the exhibition of the 1st. and 2nd. powers, and we have (1) rapid assimilative change (2) rapid propagation, and of course a great quantity of nutrient fluid is consumed, i e assimilated to germinal matter; and the germinal matter greatly increased in amount; but as it does increase in quantity it lessens in vitality, until we reach a point where both 1st. and 2nd. powers are lost, and all the masses are of one size, shape, appearance &c.; the next step is fatty degeneration, all vital force having been previously exhausted in this rapid propagation. Now to repeat. Mucus consists of *mucin* and germinal matter, the more *mucin* and the fewer the masses of germinal matter, the higher the grade, for the more formed material we find; the less the quantity of mucin and greater the number of masses of germinal matter, the lower the mucus. When therefore the germinal matter instead of exercising the 2d. exercises feebly the 3d. power we will have a high grade of mucus ;—when however the germinal matter shows the 2nd. and not the 3rd. we may expect a low grade not very far removed from *pus*; mucus and pus are but different products of the same process, they mark two stages, mucus is the beginning, pus is the end. Germinal matter arrested in its developement into Epithelial or Epidermic structures, may pass thro' the two stages and give us blennorrhœa and pyorrhœa.

We have here the explanation of the exhausting drain of mucoid discharges and suppurations, the blood furnishing the nutrient plasma, is consumed in this worse than useless manufacture of a devitalized product.

Mucus and pus upon a membrane never contribute to but always embarrass more or less the function of this membrane.

The question might here arise, as to how these substances, which at least begin near the surface of the basement membrane, reach the free Epithelial or Epidermic surfaces?

There are two ways, both are common. (1) Germinal matter possesses the power of passing directly through epithelial cells, leaving those cells unharmed, undisturbed, intact. Dividing and sub-dividing, growing and increasing, even while passing through the epithelium; upon the free surface this continues and the nutrient fluid passes through the same rout to be consumed by the germinal matter.

(2) The epithelial coat is sometimes disrupted, broken up; some cells partially disorganized, and the *debris* of this layer can be found intimately blended with the mass of mucus or pus, giving mucus and pus globules, i e masses of half vitalized germinal matter, and scales, cells and fragments of cells. Sometimes mucus and sometimes pus will raise the cuticle lying between the cuticle and basement membrane, you have seen each upon blistered surfaces. You would say then that mucus and pus are the offspring of masses of germinal matter, which from some cause have been arrested in development and left with only the 1st. and 2nd. powers, i e (1)

Assimilative change, (2) Propagation ; that in the feebleness or absence of the 3rd, these two having full sway, are capable of producing all of the phenomena seen in their formation.†

When we discuss the functions of respiration, digestion, absorption &c., we will endeavor to show what are some of the functions of mucus membrane, but we tell you now, that the production of mucus is not one of their normal functious, but an accidental complication and one to be deprecated.

---

† In the production of mucin the 3rd power is certainly exhibited, but in an abortive form.

---

